# Polyamide Surface Layer Nano-Indentation and Thermal Properties Modified by Irradiation

**DOI:** 10.3390/ma13132915

**Published:** 2020-06-29

**Authors:** Martin Ovsik, Miroslav Manas, Michal Stanek, Adam Dockal, Jiri Vanek, Ales Mizera, Milan Adamek, Pavel Stoklasek

**Affiliations:** 1 Faculty of Technology, Tomas Bata University in Zlín, Vavreckova 275, 760 01 Zlín, Czech Republic; stanek@utb.cz (M.S.); a_dockal@utb.cz (A.D.); j4_vanek@utb.cz (J.V.); 2 Faculty of Applied Informatics, Tomas Bata University in Zlín, CEBIA-Tech, Nad Stranemi 4511, 760 05 Zlín, Czech Republic; manas@utb.cz (M.M.); mizera@utb.cz (A.M.); Adamek@utb.cz (M.A.); pstoklasek@utb.cz (P.S.)

**Keywords:** polyamide, surface layer, cross-linking, electron rays, nano-indentation, gel content

## Abstract

This study describes the effect of electron radiation on the nano-mechanical properties of surface layers of selected polyamide (PA) types. Electron radiation initiates the cross-linking of macromolecules in the polyamide structure, leading to the creation of a 3D network which fundamentally changes the properties of the tested polymers. Selected types of polyamide (PA 6, PA 66 and PA 9T) were exposed to various intensities of electron radiation (33 kGy, 66 kGy, 99 kGy, 132 kGy, 165 kGy and 198 kGy). The cross-linked polyamides’ surface properties were measured by means of the modern nano-indentation technique (Depth Sensing Indentation; DSI), which operates on the principle of the immediate detection of indenter penetration depth in dependence on the applied load. The evaluation was preformed using the Oliver–Pharr method. The effect of electron radiation on the tested polyamides manifested itself in the creation of a 3D network, which led to an increase of surface layer properties, such as indentation hardness, elastic modulus, creep and temperature resistance, by up to 93%. The increase of temperature and mechanical properties substantially broadens the field of application of these materials in technical practice, especially when higher temperature resistance is required. The positive changes to the nano-mechanical properties as well as mechanical and temperature capabilities instigated by the cross-linking process were confirmed by the gel volume test. These measurements lay the foundation for a detailed study of this topic, as well as for a more effective means of modifying chosen properties of technical polyamide products by radiation.

## 1. Introduction

The effect of ionizing radiation on various polymer properties is a wide and well-researched area. Although sources focusing on the description of the process are abundant, their scope and complexity do not meet the necessary complexity threshold for a broader understanding. Furthermore, the currently available information about ionized radiation interaction with thermo-mechanical properties is insufficient, and a thorough study of this topic is required [1].

Cross-linking is a chemical process, in which ionized radiation initiates the creation of spatial structures in linear polymers. The chemical reactions lead to permanent changes in the polymer structure (spatial network). Exposure to ionized radiation is followed by the inception of active centers that react with each other [2].

**Figure 1:**
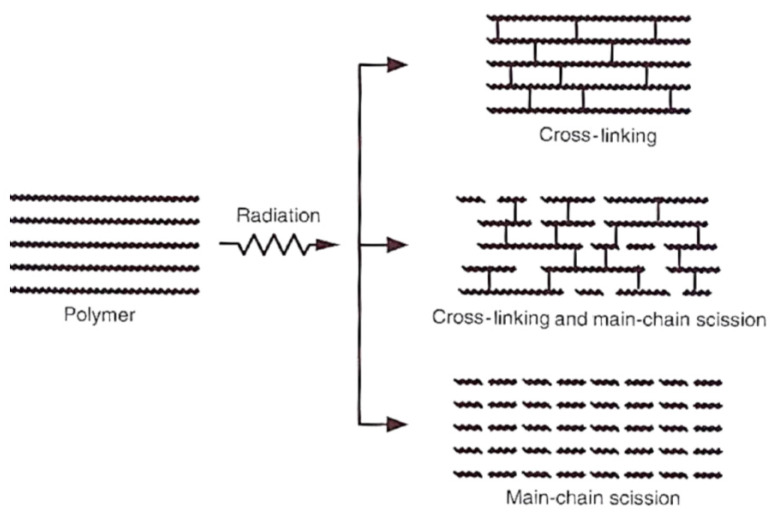
Influence of electron rays on polymers [3].

Transverse network bonds develop by the recombination of the polymer radicals, as can be seen in Figure 1. Cross-linking and degradation of the primary chain usually happen concurrently. Whenever the cross-linking process rate is two times higher than the degradation process, the structure of a polymer is cross-linked [3].

New developments and trends in the radiation technology field are summarized in several published papers, especially those written by Tabata [2], Ivanov [4], Zhang [5], and Wood and Pikaev [6]. The new trends and developments in this area are also presented in works of authors Sun [7], Lyons [8] and Chmielewski [9].

On the other hand, only a small amount of books and specialized publications focus on the industrial applications of polymers cross-linked by radiation. One of the few books that offers a more detailed look into the latest trends in the new applications field, while also providing a solid overview of current uses of radiation technology in several market areas, has been published by Makuuchi and Cheng [10].

Finally, several more studies focus on the influence of radiation cross-linking on polyamides (PA) [11,12,13,14,15,16,17,18,19,20,21,22,23], although they offer only low amounts of systematic and complex information on this topic. Hence, it is quite difficult to determine relevant correlations between ionized beta radiation and changes in the morphology with subsequent modifications to the mechanical properties’ profile required for the industrial applications of polyamides cross-linked by radiation.

In general, polyamides display medium resistance to ionized radiation. This resistance is lower in polyamides with –CONH–(CH_2_)_n_– molecule groups with the increasing number of methyl groups between the peptide bonds. As a consequence of irradiation, both the cross-linking and chain fission processes can occur, although the cross-linking process is dominant for most reactions [11]. The polyamide irradiation with oxygen present leads to the polymer chain fission and formation of peroxides as the cross-linking is not a superior process under these conditions. According to Kaindl and Graul [24], free radicals created during the irradiation process appear dominantly within the structure (1) and (2). Apart from that, the free radicals are also created within the double bonds of C=N and C=C:

(1) $$-\mathrm{CONH}-C^{*}H-{\mathrm{CH}}_{2}-$$

(2) $$-C^{*}O-\mathrm{NH}-{\mathrm{CH}}_{2}-$$

On the other hand, the application of the radiation in a vacuum leads to the dominance of the cross-linking reaction, which causes improvement in mechanical and thermo-mechanical properties. Furthermore, the addition of poly functional monomer, i.e., triallyl isocyanurate (TAIC), enables the cross-linking of polyamides at relatively low radiation dosages and with oxygen present.

The first tested polymer was polyamide 6. This hard, lightly yellow looking substance with a callous surface can transform into low viscosity liquid after passing its melt temperature range from 215 to 220 °C. Polyamide 6 is insoluble in common solvents, but it can be dissolved in phenols, formic acid and water free acetic acid. Polyamide 6 offers good mechanical properties, a low friction coefficient, extraordinary abrasion resistance, and good impact strength and electro isolation properties. Its weaknesses rest in low resistance to moisture, mineral acids and oxidation agents [25].

The next polymer examined in this study is PA 66. It is a semi-crystalline thermoplastic polymer which can offer good toughness, strength, abrasion resistant properties and a high melting temperature. On the other hand, it has minimal dimensional stability due to water retention, is high cost, and possesses a low melt viscosity threshold [26]. After the irradiation of polyamide 66 in the presence of triallyl isocyanurate as a cross-linking agent, the improvement of physical and mechanical properties, such as tensile strength, tensile modulus, flexural modulus and Rockwell hardness, was found. The cross-linked blend of polyamide 66 in the presence of TAIC and polyurethane had the highest impact resistance. Water absorption was reduced after electron beam irradiation of the samples [27].

The third thermoplastic material which is irradiated and investigated in this research paper is PA 9T. This is a semi-aromatic polyamide which contains a long flexible aliphatic linkage containing nine methylene groups incorporated in the main chain that allow one to obtain a lower melting temperature. PA 9T possesses good temperature stability, high temperature-moisture resistance, low water absorption and a good resistance to acid, alkali, organic solvents and hot water [28,29].

The goal of this study is to describe the influence of electron radiation on the mechanical and temperature stability of selected polyamides (PA 6, PA 66 and PA 9T) surface layers. Electron radiation manifests itself by the creation of 3D networks, which positively affects the final properties. The creation of the 3D network within the structure of the polyamide was confirmed by the gel content test. Due to this modification, the field of the technical application of these polymers is expanded, since they can be used to replace the more expansive polymers while providing similar properties.

## 2. Materials and Methods

The field of studies concerned with the polymer surface layer area is wide, e.g., the examination of nano-mechanical properties (DSI), thermal properties (TMA) or structural changes, (gel content). This research deals particularly with the study of nano-mechanical property changes in varying types of polyamide induced by radiation cross-linking. Selected types of polyamide were chosen out of an assortment of frequently used technical polymers commonly employed in fields like the automotive and aviation industries.

### 2.1. Materials

Choosing an appropriate material is contingent on the necessary active properties of the ultimate product. Specifically, the main intention is the introduction of some of the specialized polymer properties in polymers commonly used for manufacturing processes by the creation of a 3D network within the structure. This can be done by irradiation and it could lead to the substitution of costly materials adopted for various applications.

The presented research paper focuses on the polymer materials commonly used in technical practice (PA 6, PA 66 and PA 9T). For the study of the material properties development occurring after irradiation, polyamides were picked by the virtue of their capability to cross-link with the addition of any polyfunctional monomer (cross-linking agent) to the structure (polymer matrix).The reason for adding the cross-linking agents lies in improving the efficiency of the process that creates the 3D network. Table 1 displays the evaluated polymers.

These polymers were exposed to beta-radiation; however, a special cross-linking agent is required. TAIC (triallyl isocyanuran) in a 6% wt concentration cross-linking agent was used. The entire granulate preparation process with the cross-linking agent was performed by the PTS Plastic Technology Service company.

### 2.2. Sample Preparation

**Figure 2:**
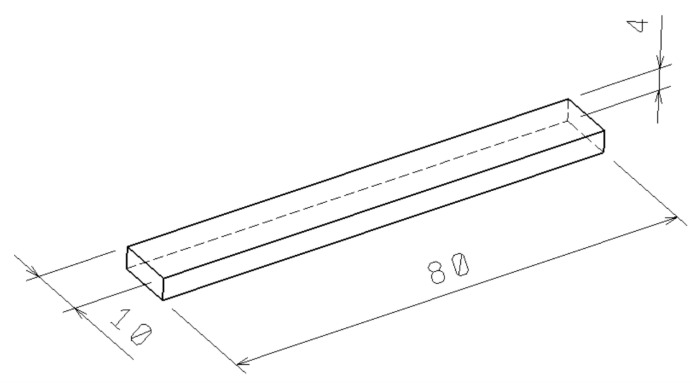
Test sample dimensions.

The preparation of the test samples was done by injection molding on an ARBURG type Allrounder 470e (Loßburg, Germany) machines. Table 2 displays the conditions which were set for the injection molding according to the manufacturer’s recommendations. The materials were dried (the drying temperature was 90 °C for 4 h) in accordance with the supplier’s guidelines in an ARBURG THERMOLIFT 100-2 drying device (Loßburg, Germany). The specimen proportions (in shape of a bar), which are shown in Figure 2, were set with respect to the CSN EN ISO 179 norm.

### 2.3. Irradiation

The tested samples were exposed to beta-radiation under normal atmospheric conditions and at room temperature, (23 °C). This modification was performed by the Beta-Gamma-Service (BGS) GmbH & Co. KG (Saalan der Donau, Germany) branch office. The radiation source was a Rhodotron 10 MeV 200 kW (Tongeren, Belgium) toroidal electron beam accelerator. The range of the dosages was set, in compliance with experience gained from industrial practice, at 33, 66, 99, 132, 165 and 198 kGy. Each accelerator cycle exposed the test sample to the radiation dose of 33 kGy. Furthermore, a dosimeter was used to measure the absorbed radiation dosage—which was also subsequently determined by employing a Spectronic Genesys 5 (Goleta, CA, USA, photometric device).

By adding a polyfunctional monomer, e.g., triallyl isocyanuran, lower radiation dosages are required to induce cross-linking in polyamides. This process takes place in a non-vacuum ambiance, where oxygen is present. Investigating the influence of the selected dosages and the evaluation of the resulting nano-mechanical properties is the main center of interest of this paper. Firstly, the hydrogen connected to the carbon neighboring with the amide group was separated, thus creating water molecules, carbon monoxide, carbon dioxide and methane. As a consequence, three TAIC allyl groups could react due to the macro radicals created by the radiation. Inevitably, they bonded in the form of bridges (networks).

### 2.4. Nano-Indentation Test

The irradiated polymer surface property measurement process employed the use of an (NHT^3^) nano-indentation tester, manufactured by Anto Paar (Graz, Austria). The test was complied with the CSN EN ISO 14,577 standard. The principle of the instrumented hardness tests is in continuous recording of the loading Force “P” depending on the instantaneous penetration depth “h” of the indenter. This dependence is graphically recorded as a so-called indentation curve. The Oliver and Pharr method [30,31] was used to evaluate the measured data. This method makes it possible to determine the values of indentation hardness, modulus and creep.

The penetrating body was a Berkovich indenter. Table 3 displays the process parameters used.

**Figure 3:**
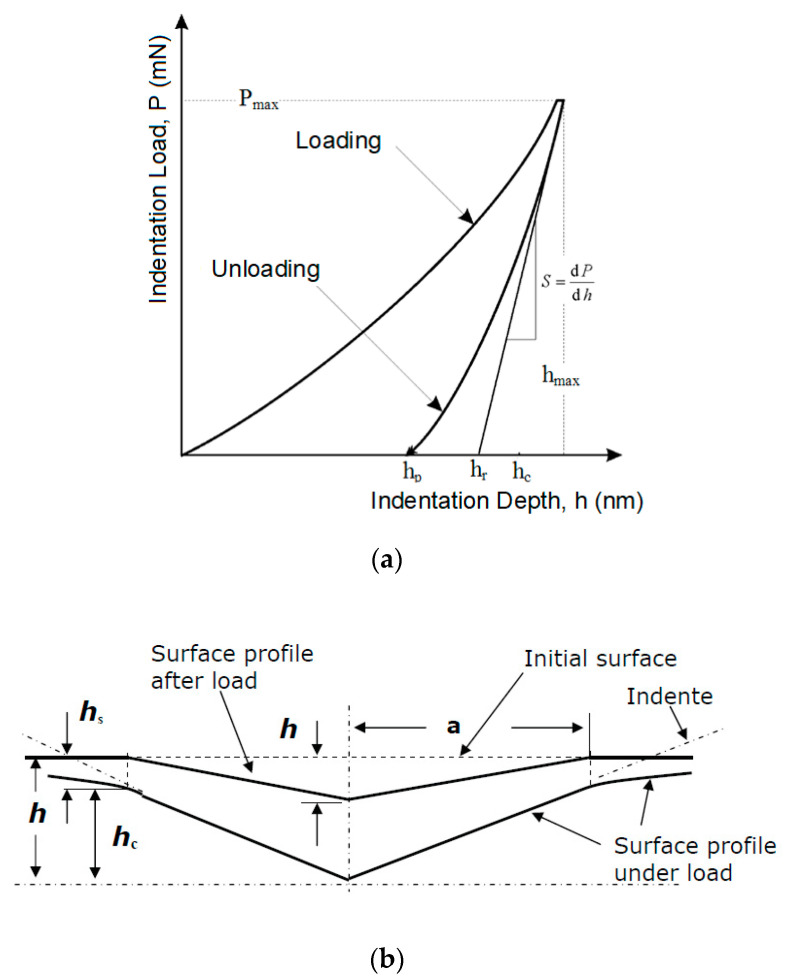
Indentation profile: (**a**) load–displacement curve [30]; (**b**) cross-section of an indentation [30].

Indentation hardness (*H_IT_*) was calculated as the maximum load (*F_max_*) on the projected area of the hardness impression (*A_p_*) (Figure 3) [30,31]:

(3) $$H_{IT}=\frac{F_{max}}{A_{p}}$$

(4) $$A_{p}=23.96\times h_{c}^{2}$$

The indentation modulus (*E_IT_*) was calculated from the plane strain modulus (E*), using an estimated Poisson’s ratio (*ν_s_*) sample—(Polymer 0.3 to 0.4) [30,31,32]:

(5) E=ITE*×(1−vs2)

(6) $$E^{*}=\frac{1}{\frac{1}{E_{r}}-\frac{1-v_{i}^{2}}{E_{i}}}$$

(7) $$E_{r}=\frac{\sqrt{\pi }}{2\times C\sqrt{A_{p}}}$$

where *E_i_* is the elastic modulus of the indenter (diamond 1141 GPa), *E_r_* is the reduced indentation contact modulus, and *ν_i_* is the indenter Poisson’s ratio (0.07).

**Figure 4:**
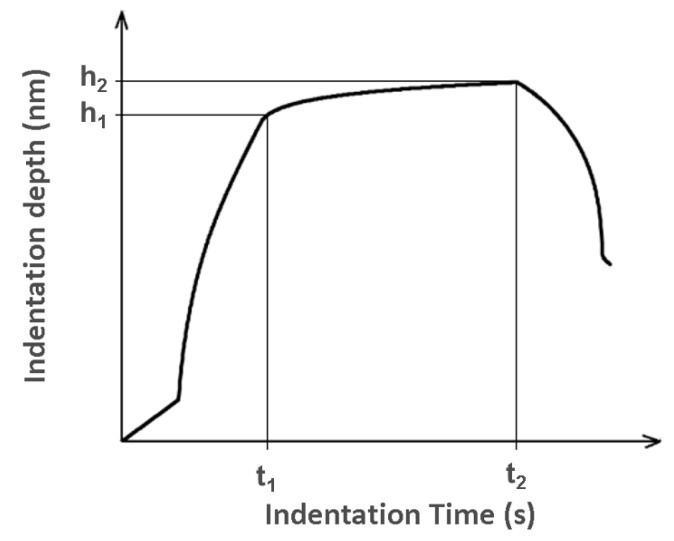
Displacement–time curve showing the indentation creep.

The determination of indentation creep C_IT_ (where h_1_ is the indentation depth at time t_1_ of reaching the maximal test force) was as follows—h_2_ is the indentation depth at time t_2_ of withstanding the constant test force (Figure 4) [30]:

(8) $$C_{IT}=\frac{h_{2}-h_{1}}{h_{1}}\times 100\%$$

### 2.5. Gel Content

Linear polymers are produced by the chemical linkage of two polymer functional groups. Furthermore, if one of the groups is multifunctional, a swelling and elongation with branching can be observed. This leads to the creation of an infinite 3D network, i.e., a gel.

There are cross-linking actions that happen sequentially in all cases [1]:

- Dimensions and polydispersion grow in the first phase.
- At a certain point, the reaction reaches a gel point in which the molar weight increases beyond all limits and the gel starts to appear in the system.
- After bypassing the gel point, the system is composed of two parts: on the one hand, an infinite structure called a “gel”; on the other, a cluster of molecules with finite size called a “sol”. These molecules can be separated from the gel by extraction.
- Complete networks, by-products and micro-gel cannot be extracted. The gel is insolvable and if exposed to a solvent, it experiences imbibitions.
- Finally, the sol content, as well as its molar weight and polydispersity, decreases as the reaction continues.
- Active elastic chains are created within the gel network. These chains support the applied stress, thus determining the gel elastic modulus value and its equilibrium degree imbibitions.

A gel (content) test was performed in order to determine the insolvable gel content of the given material. This was in accordance with the ASTM D 2765 standard—Test Method C. A portion of 0.5 g, weighed with a precision of five decimal places was mixed with 100 mL of solvent on a “SWISS MADE EP 125 SM” weighing apparatus (Dietikon, Switzerland). Xylene was used as the solvent for the tested polymers because it dissolves the amorphous part of the material, while the cross-linked part remains intact. The mixture’s extraction duration was 24 h. Then, the solutes were separated by distillation. After removing the residual xylene, the cross-linked extract was dried for 8 h, in a vacuum, at 100 °C. The dried and cooled residue was weighed again with a precision of five decimal places and compared to the original weight of the sample [1]. The result is stated in percentage as the degree of cross-linking:

(9) $$G_{i}=\frac{m_{3}-m_{1}}{m_{2}-m_{1}}\times 100\%$$

where, *G_i_* is the degree of cross-linking of each specimen expressed in percent; *m*_1_ is the weight of the cage and lid in milligrams; *m*_2_ is the total weight of the original specimen, cage, and lid in milligrams; and *m*_3_ is the total of the weight of the residue of specimen, cage and lid in milligrams.

### 2.6. Thermo-Mechanical Analysis (TMA)

Temperature stability was assessed using a thermo-mechanical analysis (TMA) in the penetration mode. The thermo-mechanical properties were measured using a Perkin-ElmerDMA 7e thermal analyzer (Waltham, MA, USA) which was used for the thermo-mechanical analysis, heated from 50 to 400 °C (depending on the material used) at 20 °C/min, and held for 1 min at 50 °C. This precise temperature resistance evaluation of the polymers (e.g., irradiated, cross-linked polymers) records the displacement of the probe with a loading of 160 mN, which penetrates into the heated material, in a set range of temperatures [1].

## 3. Results

The nano-mechanical properties of the selected polymers’ surface layers were measured using the Depth Sensing Indentation (DSI) method, which is based on the principle of detecting the immediate depth of irradiation vs. the loading force at an exact point in time. Radiation cross-linking also broadens the application field of these polymers where increased temperature is considered. These properties were measured by thermo-mechanical analysis. A gel content test was conducted in order to confirm the mechanical and thermal changes as well as to determine the amount of cross-linked structure.

The resulting data was composed of ten measurements for each property, which were used to calculate the arithmetic mean and the standard deviation.

### 3.1. Nano-Mechanical Properties (Indentation Depth, Hardness, Modulus and Creep)

**Figure 5:**
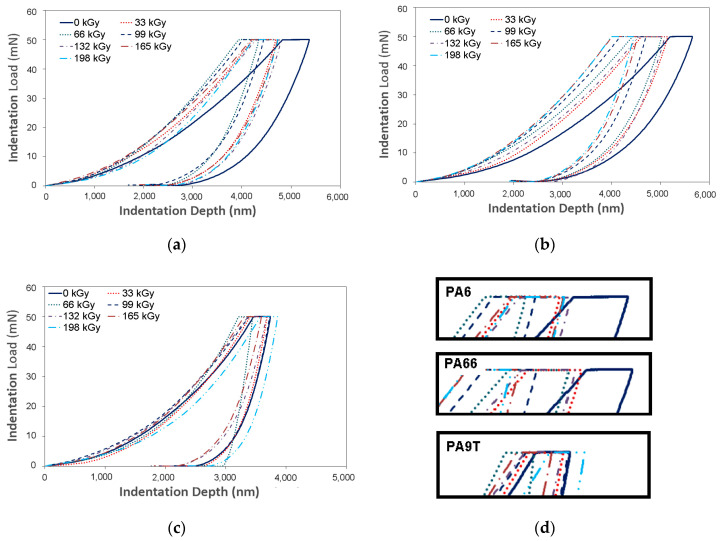
Indentation curve: (**a**) polyamide PA 6; (**b**) PA 66; (**c**) PA9T; (**d**) detail.

The basic principle of the DSI method is the instantaneous detection of indentation depth dependent on the applied load in time. The graphical representation of the indentation progress (applied load vs. indentation depth) is the curve shown in Figure 5. This curve serves to calculate the selected polymers’ mechanical properties.

The first phase of the indentation is the application of stress. During this phase, the indentation device drives into the test sample with a preset force. The second cycle phase is labeled as de-loading and it is composed of a gradual decline of the stress straight to zero. Usually, a delay exists between the previously mentioned phases. This delay is composed of exposing the specimen to the maximum force, which allows the measurement of the indentation creep.

**Figure 6:**
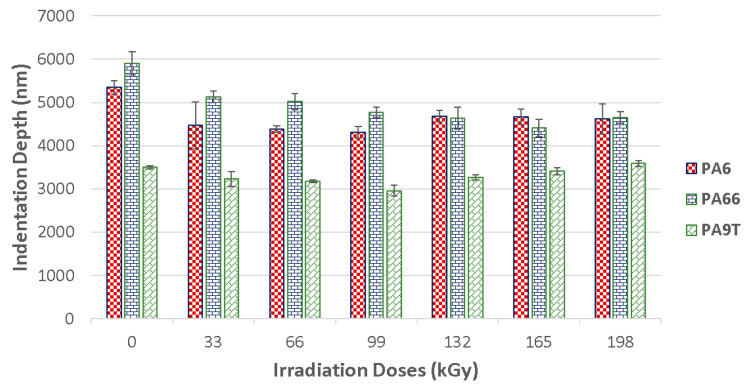
Indentation depth (h_max_) of irradiated PA 6, PA 66 and PA 9T.

The maximum depth reached during the measurement of the mechanical properties is an important parameter that provides information about the surface layer (Figure 6). The maximum indentation depth was 5.400 nm for virgin PA 6, 5.800 nm for virgin PA 66, and 3.500 nm for virgin PA 9T. Every other specimen displayed a lower penetration depth. However, this enables the measurement of the surface layers properties of the tested polymer.

**Figure 7:**
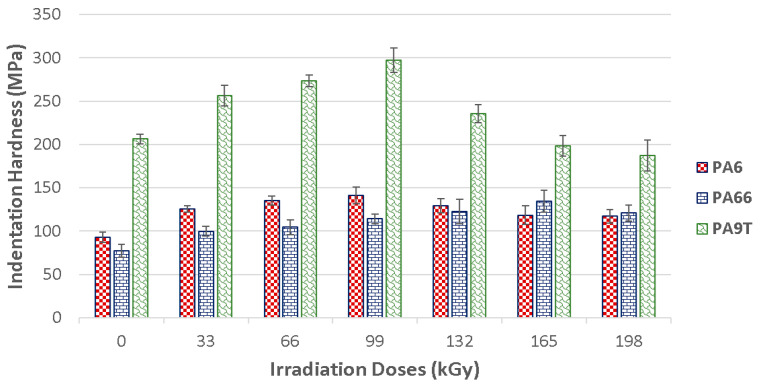
Indentation hardness (H_IT_) of irradiated PA 6, PA 66 and PA 9T.

Indentation hardness (H_IT_) is the degree of material resistance to permanent deformation or damage. Figure 7 shows graphical representation of the indentation hardness in dependence on the varying radiation dosages. The results indicate that the radiation cross-linking of the tested materials increases their surface layer hardness.

PA 6 can be cross-linked by beta radiation with the help of a cross-linking agent. TAIC (triallyl isocyanuran), in 6% volume concentration, was used as the cross-linking agent. These test samples displayed an increase in indentation hardness after being exposed to beta radiation. The biggest indentation hardness values (141 MPa), were found in these specimens, exposed to 99 kGy of radiation. The unaltered material indentation hardness was 93 MPa. Due to the creation of the 3D network caused by the irradiation, the indentation hardness rose by 52% in comparison to the unaltered material. On the other hand, the application of more than 99 kGy of radiation proved to stabilize the indentation hardness values. 

Similar tendencies could be seen in the PA 9T test samples, in which the virgin material displayed an indentation hardness of 206 MPa. The highest indentation hardness values (297 MPa) of the polyamide was measured after its exposure to a dosage of 99 kGy. The indentation hardness rose by 44% due to this gradual irradiation. With each added level of radiation intensity, the indentation hardness values haltingly decreased all the way down to the virgin material values. The test samples’ indentation hardness was 187 MPa at a radiation dosage of 198 kGy. This decrease was caused by the degradation of the surface layer, induced by the high intensity radiation.

**Figure 8:**
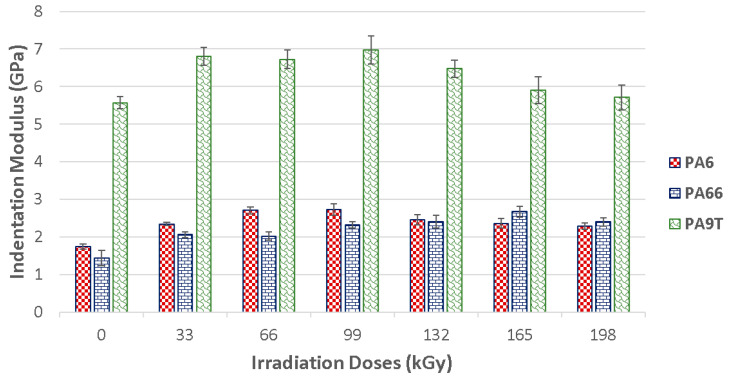
Indentation modulus (E_IT_) of irradiated PA 6, PA 66 and PA 9T.

Radiation cross-linking was also found to have a significant effect on the nano-mechanical surface layer properties of PA 66. The base material displayed an indentation hardness of 77 MPa, while the irradiated material indentation hardness gradually rose to 134 MPa, which was the peak value after the test sample was exposed to a radiation dosage of 165 kGy. The irradiation of the polyamide led to a 74% indentation hardness increase with respect to the unaltered material. Dosages higher than 165 kGy showed the stabilization of the indentation hardness values. As can be seen in Figure 8, the indentation hardness of the tested construction polymers is strongly affected by the cross-linking process. The decrease of indentation hardness values that occurred when the specimens absorbed more than 99 kGy of radiation for PA 9T could have been caused by material degradation generated by the irradiation.

The indentation modulus was positively affected by radiation cross-linking in all of the polymer types. On the one hand, PA 6 irradiated by lower levels of radiation showed improved values for the indentation modulus, while showing declining values for higher radiation values. These displayed a gradual increase in indentation modulus for the 99 kGy radiation dosages. 

The biggest values of indentation modulus were found in specimens exposed to 99 kGy of radiation—this was 2.7 GPa. The indentation modulus increased by 56% with respect to the unaltered material, all due to their exposure to radiation. With an intensity of more than 99 kGy of radiation, the material has shown a stabilizing tendency of the indentation modulus values.

In contrast, the effect of the radiation was more pronounced for PA 66. The indentation modulus of the virgin material was 1.4 GPa, and gradual exposure to radiation increased it to 2.7 GPa for a dosage of 165 kGy. So, the indentation modulus was raised by 93% in comparison to the unaltered material. 

The change of the surface layer indentation modulus induced by radiation cross-linking in high tech polymers, like PA 9T, was also observed. The indentation modulus values gradually rose with each added radiation level up to a dosage of 99 kGy. The indentation modulus was 25% higher in comparison to unaltered PA 9T. With dosages higher than 99 kGy, the material exhibited a decrease of indentation modulus values, all the way down to the level of the virgin material.

**Figure 9:**
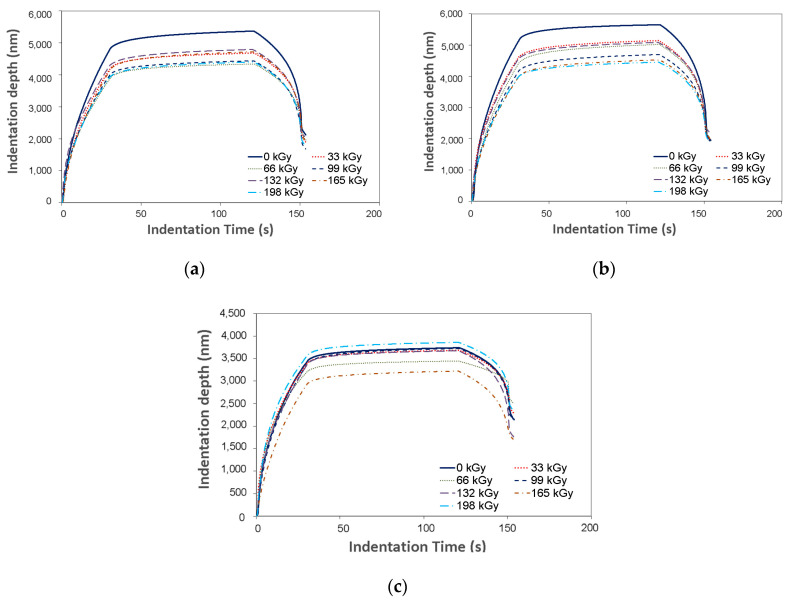
Indentation creep (indentation depth vs. indentation time): (**a**) PA 6; (**b**) PA 66; (**c**) PA 9T.

Figure 9 displays the tested polymers’ properties before and after the application of varying radiation dosages. Another important polymer surface layer parameter is the indentation creep that occurs when the material is exposed to constant stress. In technical practice, this can occur quite often. Measuring the indentation depth while applying constant stress, the relative indentation depth can be calculated. The values of the relative indentation depth are the same as the values of the material creep. Figure 9 provides the meaningful development of the properties which were measure after the individual radiation dosage exposure happened.

**Figure 10:**
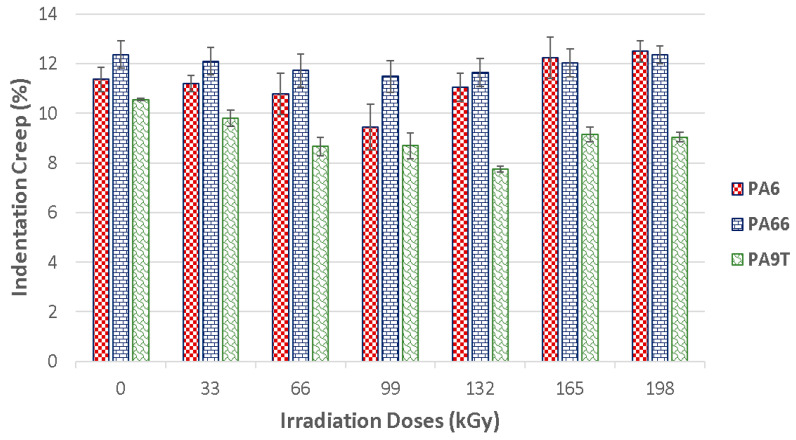
Indentation creep (C_IT_) of irradiated PA 6, PA 66 and PA 9T.

The results clearly show that the irradiation of the specimen has a positive effect on material creep properties (Figure 10). The creep results concur with the indentation hardness and indentation modulus results.

The best material creep resistance was measured in PA 6—irradiated by 99 kGy. The difference in comparison to the unaltered material was 20%. Similar results were measured for PA 66, where the best creep resistance was also found in the specimen exposed to 99 kGy. The improvement in relation to the virgin material was lower than that of PA 6, since it was 8%. The best PA 9T creep resistance was found in the test sample exposed to 132 kGy. Creep resistance improved by 36% in comparison to the unaltered material.

As is evident from the indentation creep measurements, ionized radiation positively influences the resistance of the tested materials to creep in time. This can lead to better material usage when exposed to long-term stress.

The surface layer mechanical properties measurement results show that the application of electron radiation leads to substantial improvements in useful properties, which could promote broader utilization of these polymers in the practical field. These materials’ properties can be partially or fully extrapolated to more expensive polymers, and in doing so, replace them. 

### 3.2. Thermo-Mechanical Analysis

Thermo-mechanical analysis was another important mechanical analysis used to understand the influence of ionized radiation on selected polymers. The TMA results correspond to the nano-mechanical properties’ results. This could lead to material applications in more challenging situations where higher surface resistance is required during exposure to higher temperatures.

**Figure 11:**
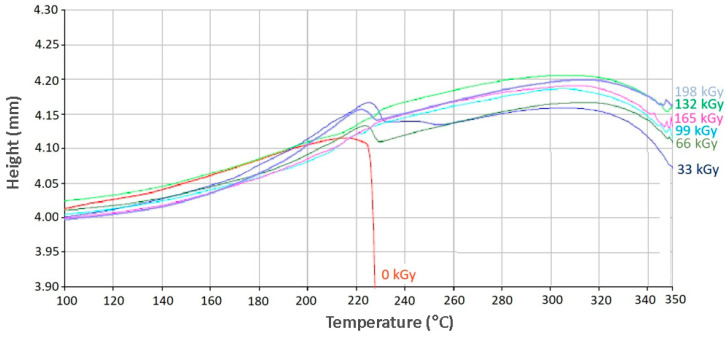
Thermo-mechanical analysis (TMA) (PA 6).

As can be seen in Figure 11, virgin PA 6 is stable up to its melt-temperature, which is approximately 220 °C. Further temperature increases lead to the probe’s penetration into the material. The effect of radiation dosages leading to the creation of such a spatial network, predominantly in an amorphous region, cause the material to lose its plasticity, while showing lower deformation when exposed to constant load in dependence on temperature. This temperature was in the melt temperature area of unaltered material, and the measurements showed that selected polymers were stable up to a temperature of 340 °C by an irradiation dose higher than 66 kGy. Increasing the temperature beyond 340 °C led to the pyrolytic thermo-oxidative degradation of the specimen.

**Figure 12:**
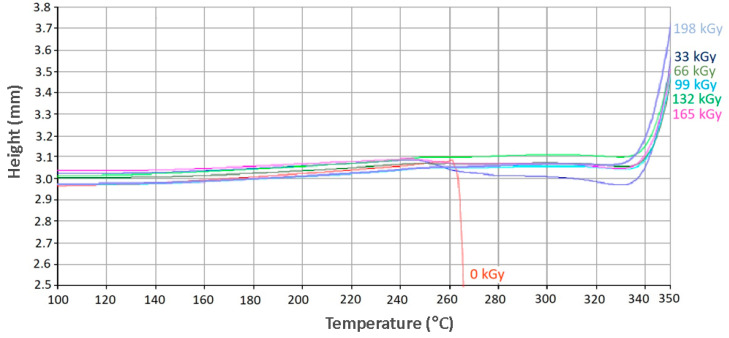
Thermo-mechanical analysis (PA 66).

Tendencies similar to PA 6 can also be observed in PA 66, in which the unaltered specimens were stable up to a melt temperature of 260 °C. Further temperature increases led to the melting of the material. Every other sample exposed to an irradiation dose higher than 66 kGy proved to be heat stable up to a temperature of 340 °C, as can be seen in Figure 12.

PA 9T is a polymer designed for use at higher temperatures (200 °C and above) while maintaining the same properties as at room temperature. PA 9T is thermoplastic polymer designed for special applications in the automotive and aerospace industries. It is usually reinforced with a high content of fillers such as carbon or glass filers. PA 9T can be further modified to improve its temperature stability and mechanical properties. Frequently used modifications include, in particular, radiation cross-linking.

**Figure 13:**
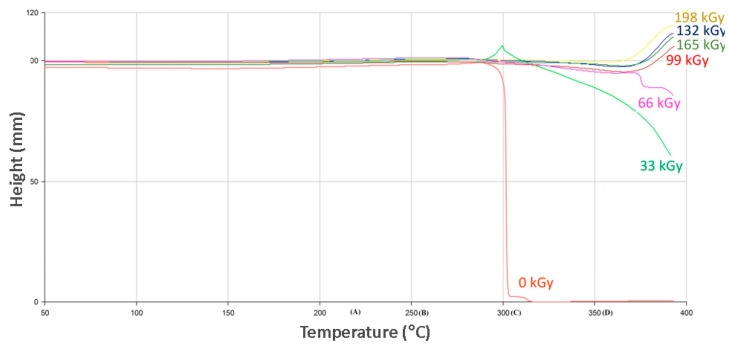
Thermo-mechanical analysis (PA 9T).

The evaluation of the thermal and mechanical properties of PA 9T were done by TMA measurement in penetration mode. The TMA evaluation shows that the unaltered specimens quickly melt at the temperature of 300 °C (as can be seen in Figure 13). The measurements show that the PA 9T specimens exposed to radiation had improved temperature stability, even though the recorded changes were minimal. As can be also seen in Figure 13, the irradiated PA 9T polymer can be used in environment with ambient temperature higher than the melting point of the virgin polymer. Products made from altered PA 9T, cross-linked by a radiation dose of 66 kGy, could work at 350 °C for a certain time without fatal damage.

The measurements show that the structural changes initiated by various dosages of ionized beta radiation have a positive influence on overall temperature resistance in all tested irradiated polymers.

The measurement results show that suitable radiation dosages for all tested specimens intended for use in higher temperature environments was 66 or more kGy. The enacted TMA measurements and visual comparison proved the improvement of temperature resistance when exposed to higher dosages of radiation. On the other hand, it is always important to individually assess every aspect of the process, including irradiation costs.

The results of temperature resistance are in accordance with the findings of other authors, e.g., Brocka [11].

### 3.3. Gel Content

**Figure 14:**
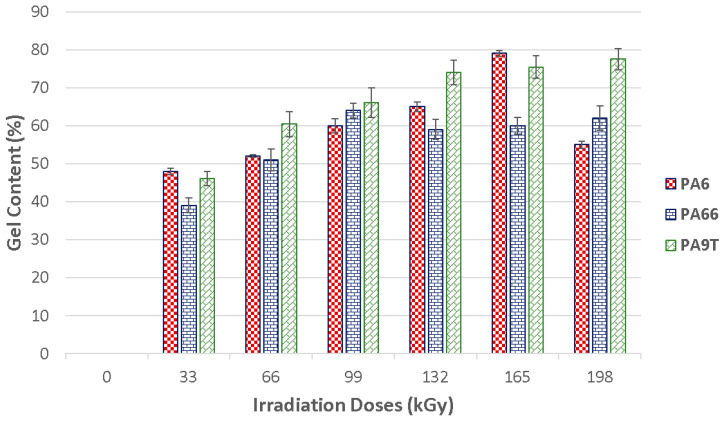
Gel content of irradiated PA 6, PA 66 and PA 9T.

The gel content test was conducted in order to measure the phase or gel of the specific material nonfiltered volumes according to the EN ISO 579 standard. The determination of gel content in the selected polymers dependent on the applied radiation dosage can be seen in Figure 14.

As indicated in Figure 14, enhancement occurs even with the lowest radiation amount—which was 33 kGy. The virgin construction materials (PA6 and PA 9T) were completely dissolved during the gel test, which confirms the previous test findings that indicated the lowest nano-mechanical properties’ values in these test samples. A meaningful increase in gel content was observed even in materials exposed to the lowest amounts of radiation (33 kGy). Moreover, the gel content was gradually increased with the higher radiation intensity.

For the PA 6 test samples, the maximum gel content value was measured in subjects irradiated by a dosage of 165 kGy. These findings are in agreement with the nano-mechanical results, in which the highest indentation hardness value was measured in the similarly altered materials. For PA 66, the highest gel content amount was measured in test samples irradiated by a radiation dosage of 99 kGy, which also corresponded with the maximum nano-mechanical properties values. The gel content experienced a minor fall when exposed to higher radiation values, and this was also in agreement with the surface layer nano-mechanical properties.

As is evident from Figure 14, the degree of cross-linking, i.e., gel content, had an effect on every tested samples’ properties. For PA 9T, the gel content gradually rose and reached the maximum in all samples irradiated by a radiation dosage of 198 kGy.

## 4. Conclusions

This study describes the influence of varying electron radiation dosages on the nano-mechanical and thermal properties of the tested polyamides. The results show that electron radiation positively affects the mechanical and thermal properties of all tested polymers. These modifications to these properties differed not only by various radiation dosages, but also by different materials.

The specimens were chosen from the construction materials field, which has good application potential in technical practice. This group consisted of Polyamide 6, Polyamide 66 and Polyamide 9T. These materials were exposed to diverse electron radiation intensities (33 kGy to 198 kGy), which supported the creation of 3D networks within their structure, leading to distinctive changes in the tested materials.

Electron radiation had a positive effect on nano-mechanical properties in all materials. The optimal radiation dosage for PA 6 was 99 kGy, which increased its indentation hardness by 52%, the indentation modulus by 56%, and indentation creep by 20% in comparison to the virgin material. The best radiation dosage for PA 66 appears to be 165 kGy, which increased indentation hardness by 74%, the indentation modulus by 93%, and indentation creep by 8% in comparison to the virgin material. The optimal radiation dosage for PA 9T was 99 kGy, which increased indentation hardness by 44%, the indentation modulus by 4%, and indentation creep by 36% in comparison to the virgin material. Higher radiation dosages did not always mean significant improvement of the tested properties, which could have been caused by degradation processes induced by high radiation intensities.

The thermo-mechanical analyses results proved the essential influence of ionized radiation dosages on the tested polyamide temperature resistance. It has been shown that this modification broadens the application potential of polyamide products in the field of functional parts exposed to higher working temperatures. These temperatures even reached the values of highly temperature resistant thermoplastics. This finding adds value to polyamides cross-linked by radiation, while decreasing overall cost.

Changes initiated by electron radiation were confirmed by gel content tests that proved the creation of cross-linked parts in these structures—these treated parts positively and substantially influence their mechanical and thermal properties. The gel content test results confirm the nano-mechanical and thermal properties data that were measured.

The final results of the measurements could have been influenced by numerous factors. Nevertheless, it is feasible to state that the possibility of polyamide modification by ionized radiation is a real option leading to a positive effect on irradiated material properties. However, it is always important to individually consider a radiation dosage with respect to the required properties of the part, in a way that increases the highest possible added value.

## Figures and Tables

**Table 1:** Tested polymers.

Type of Polymers	Trade Name	Company
Polyamide 6	PA 6 (V-PTS-CREAMID-B3H2*M800/14 natur)	PTS (Adelshofen, Germany)
Polyamide 66	PA 66 (V-PTS-CREAMID-A3H2*M710 A/14 natur)	PTS (Adelshofen, Germany)
Polyamide 9T	PA 9T (V-PTS-DURAMID-9TH2G9*M800/13 natur)	PTS (Adelshofen, Germany)

**Table 2:** Process parameters.

Process Parameter	PA 6	PA 66	PA 9T
Injection rate (mm/s)	50	50	50
Injection pressure (MPa)	80	80	170
Cooling time (s)	20	20	25
Mold temperature (°C)	90	90	140
Holding time (s)	20	20	15
Barrel temperature—Zone 1 (°C)	220	245	295
Barrel temperature—Zone 2 (°C)	230	260	305
Barrel temperature—Zone 3 (°C)	245	275	315
Barrel temperature—Zone 4 (°C)	255	285	325

**Table 3:** Equipment settings.

Parameters	Unit	Value
Maximum load	mN	50
Load/unload speed	mN/min	100
Holding time (*H_IT_*, *E_IT_*)	s	90
Holding time (*C_IT_*)	s	21,600
